# Diagnostic efficacy of magnifying endoscopy with blue laser imaging for laryngopharyngeal reflux

**DOI:** 10.3389/fmed.2025.1642702

**Published:** 2025-09-24

**Authors:** Rong Wang, Min Yu, Chaoyuan Chen, Xi Chen, Yongxiu Lin, Jianzhen Li, Gang Liu, Huan Huang, Dazhou Li, Wen Wang

**Affiliations:** ^1^Fuzong Clinical Medical College of Fujian Medical University, Fuzhou, China; ^2^Department of Gastroenterology, The Affiliated People’s Hospital of Fujian University of Traditional Chinese Medicine, Fuzhou, China; ^3^Department of General Medicine, Qiaoying Subdistrict Health Service Center of Jimei District, Xiamen, China; ^4^Department of Otolaryngology, The Affiliated People’s Hospital of Fujian University of Traditional Chinese Medicine, Fuzhou, China

**Keywords:** blue laser imaging, magnifying endoscopy, laryngopharyngeal reflux, GERD (gastroesophageal reflux disease), endoscopic diagnosis

## Abstract

**Objectives:**

We aimed to analyze the utility of magnifying endoscopy with blue laser imaging (ME-BLI) in diagnosing laryngopharyngeal reflux (LPR).

**Methods:**

The study enrolled 106 patients based on LPR-related symptoms. Using the reflux symptom index (RSI) and reflux finding score (RFS) scales as the clinical reference standard, the study cohort comprised 68 patients with LPR (RSI >13 and/or RFS >7) and 38 controls (RSI ≤13 and RFS ≤7). All participants underwent upper gastrointestinal endoscopy with ME-BLI. The patients were classified into Grades 1–4 based on the pharyngolaryngeal appearance under ME-BLI and the visibility and characteristics of intraepithelial papillary capillary loops (IPCLs) in the pharyngolaryngeal mucosa. Grades 3 and 4 were defined as LPR-positive. The diagnostic performance of ME-BLI for LPR was compared to the RSI/RFS criteria.

**Results:**

Compared with the RSI/RFS clinical reference standard, ME-BLI demonstrated a sensitivity of 89.71% (95% CI: 81.54–94.44%), a specificity of 73.68% (95% CI: 59.72–84.03%), a positive predictive value of 85.92% (95% CI: 76.34–92.04%), and a negative predictive value of 80.00% (95% CI: 64.06–90.04%) for LPR diagnosis. It also showed good consistency with RSI/RFS diagnosis (Kappa = 0.65, 95% CI: 0.52–0.78, *p* < 0.001). Good interobserver agreement in ME-BLI grading was noted (ICC = 0.858, *p* < 0.001).

**Conclusion:**

LPR has characteristic pharyngeal manifestations. ME-BLI could potentially improve LPR diagnostic accuracy; however, further validation is required.

## Introduction

Laryngopharyngeal reflux (LPR) refers to a series of symptoms and signs caused by the reflux of gastric contents into the laryngopharynx (1). Common LPR symptoms, such as chronic cough, frequent throat clearing, and pharyngeal foreign body sensation, significantly impact the patient’s quality of life. LPR is characterized by the reflux of gastric contents above the upper esophageal sphincter, resulting in extensive laryngopharynx inflammation (2). This inflammation leads to changes in the superficial microvessels, including vascular hyperplasia and vasodilation.

The reflux symptom index (RSI) (3) and reflux finding score (RFS) (4), developed by Belafsky et al., are based on the symptoms and laryngoscopy findings in patients with LPR. An LPR diagnosis is typically considered positive when the RSI is >13 points and/or the RFS is >7 points. This approach is widely recognized internationally. While the gold-standard diagnostic tool is 24-h multichannel intraluminal impedance combined with pH (MII-pH) monitoring (5), its cost and invasiveness limit its widespread clinical use.

Blue laser imaging (BLI) is an electronic chromoendoscopy technique (6). While sharing the fundamental principle of hemoglobin absorption for vascular enhancement with narrow-band imaging (NBI), BLI uses a distinct blue laser wavelength of 410 ± 10 nm (compared to NBI’s 415 ± 30 nm). This specific wavelength is selectively absorbed by deoxyhemoglobin in superficial vessels, rendering intraepithelial microvasculature dark brown/black against a light pink mucosa, thereby optimizing surface contrast. For deeper tissue penetration, a 450 ± 10 nm blue-violet laser (compared to NBI’s 540 ± 30 nm) is used to visualize the submucosal vessel architecture. BLI provides superior brightness and significantly longer observable distances than NBI. When combined with magnifying endoscopy (ME), BLI can help visualize subtle changes in mucosal microvessels. ME-BLI is commonly used to detect morphological changes in mucosal microvessels and diagnose early gastrointestinal tumors (7, 8). However, to date, no studies have reported using ME-BLI for LPR diagnosis. We hypothesized that repeated stimulations from laryngopharyngeal reflux may induce mucosal vascular changes in the laryngopharynx and that ME-BLI could detect these changes to diagnose LPR. Therefore, this study aimed to evaluate the accuracy of ME-BLI for LPR diagnosis.

## Methods

This prospective diagnostic study adhered to the guidelines of the Declaration of Helsinki and was approved by the Biomedical Ethics Committee of the 900th Hospital of the Chinese People’s Liberation Army Joint Logistic Support Force, Fuzhou, China (No. 2022-021). All participants were informed of the study’s objectives and procedures and provided written informed consent. Patients aged 18–70 years with LPR symptoms were consecutively recruited at the gastroenterology outpatient clinic between July and December 2022. Symptoms included one or more of the following: a pharyngeal foreign body sensation, chronic cough, throat clearing, hoarseness, dysphagia, abundant expectoration, nasal reflux, dyspnea, heartburn, chest pain, and stomach pain. We excluded patients with any of the following characteristics: a history of head and neck malignancy, surgery, radiotherapy, or trauma; a history of acute upper respiratory tract infection in the past month; allergic diseases; a history of prolonged smoking or heavy drinking; use of drugs that could interfere with test results within the past week, including proton pump inhibitors, potassium-competitive acid blockers, H2 receptor antagonists, and/or prokinetic drugs; or an inability to undergo gastroscopy due to serious illness or other reasons.

**Figure 1:**
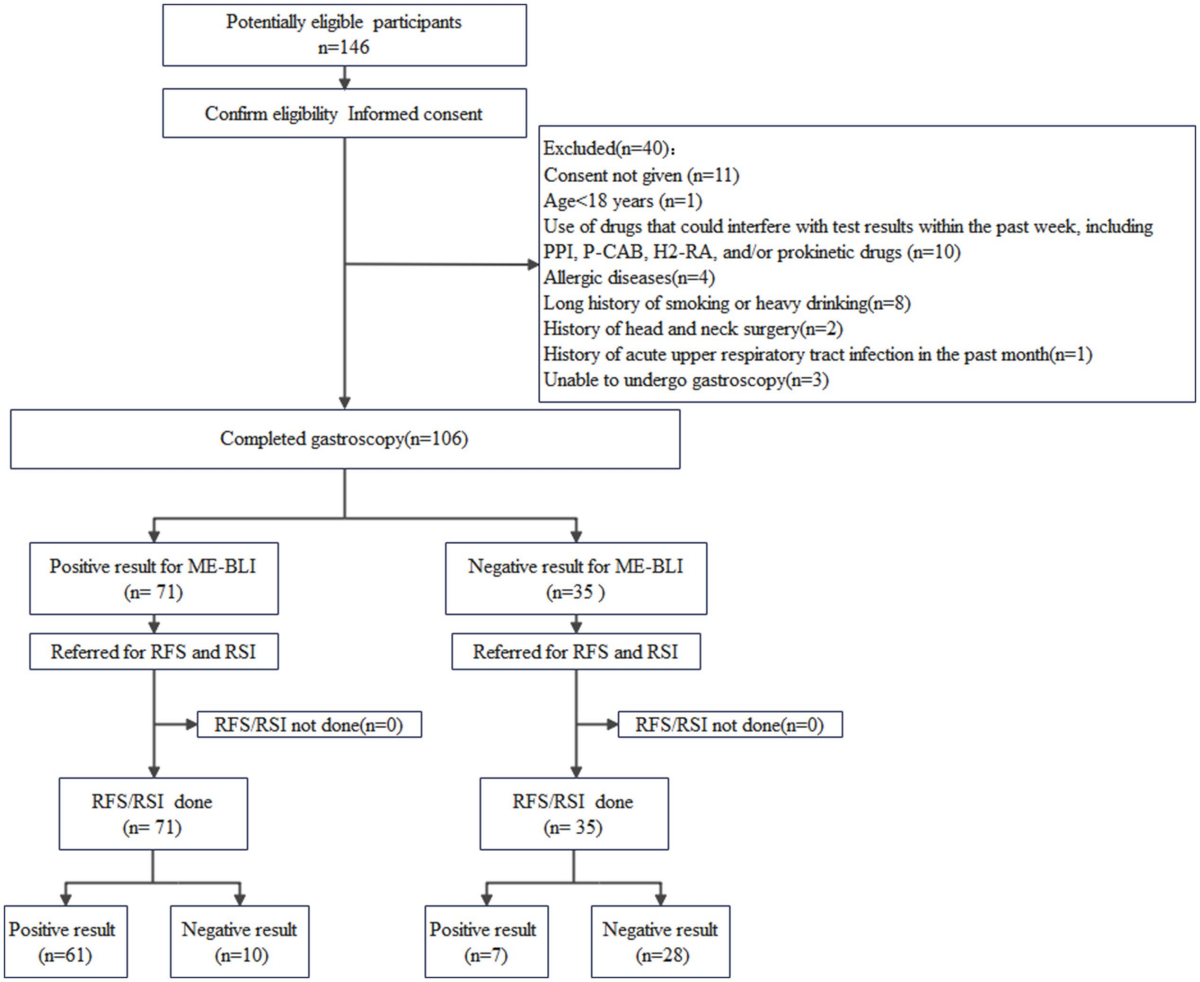
A flowchart of the participant selection process. ME-BLI, magnifying endoscopy with blue laser imaging; RSI, reflux symptom index; RFS, reflux finding score.

All patients completed the RSI questionnaire and subsequently underwent upper gastrointestinal endoscopy using the EG-L600ZW7 endoscope (Fujifilm, Xuzhou, Jiangsu, China). This endoscope allows for rapid switching between white light imaging and ME-BLI. Patients were positioned in the left decubitus position and were sedated with intravenous sufentanil (Renfu Pharmaceutical, Yichang, Hubei, China) and midazolam (Nhwa Pharmaceutical, Xuzhou, Jiangsu, China). The larynx, esophagus, stomach, and duodenum were examined successively. Each part was examined twice, first using the white light mode, and then with ME-BLI. The entire inspection process was video-recorded, and images were saved as indicated. The RFS was determined based on the laryngopharyngeal appearance in the white light mode. This composite score was based on eight evaluated items (4). The laryngeal vascular pattern was analyzed and classified according to Arens et al. (9) and Ni et al. (10). Similar to the assessment of pharyngeal inflammatory lesions by NBI, we used ME-BLI to classify pharyngolaryngitis into four grades based on the morphology of the intraepithelial papillary capillary loops (IPCLs). Grade 1, no IPCLs were detected, and intramucosal vessels were clear and exhibited no hyperplasia; Grade 2, the IPCLs were almost invisible, while intramucosal vessels were increased, dilated, or convoluted; Grade 3, the IPCLs were visible. They were regularly arranged at a sparse density and were characterized by scattered brown spots in the post-cricoid region or the arytenoid and inter-arytenoid areas; Grade 4, the IPCLs were visible. They were regularly arranged, with slightly increased density and mild dilation, and were characterized by generous tufted brown spots in the post-cricoid region or the arytenoid and inter-arytenoid areas. Two experienced chief endoscopists, blinded to the patients’ RSI scores and clinical information, underwent training in professional scoring standards before independently grading the ME-BLI and assigning the RFS scores by analyzing the images and videos. Interobserver agreement was analyzed. If the two endoscopists reached different conclusions, a third experienced chief endoscopist made the final decision. The study included 106 patients who were classified based on their RSI/RFS scores into the LPR (RSI >13 and/or RFS >7; *n* = 68) and non-LPR (RSI ≤13 and RFS ≤7; *n* = 38) groups (Figure 1).

The sample size in this pilot study was not strictly estimated; however, it meets the sample size requirement of the general empirical standard for diagnostic tests (more than 100 cases). Data were analyzed using IBM SPSS Statistics for Macintosh, Version 25.0 (IBM Corp., Armonk, NY, United States). Categorical variables are presented as frequencies and percentages (%), and continuous variables are presented as means ± SDs. Continuous variables were compared using Student’s *t*-tests, while categorical variables were compared using *χ*^2^ tests. Kappa statistics were used for the consistency test, and sensitivity, specificity, positive predictive value, and negative predictive value were used to evaluate diagnostic efficacy. Interobserver consistency was analyzed using the intraclass correlation coefficient (ICC).

## Results

### Participant characteristics

The study included 68 patients with LPR and 38 negative controls (Table 1). The groups were comparable in age, sex, and body mass index (*p* > 0.05).

**Table 1:** Clinical characteristics of the LPR and non-LPR groups.

Characteristics	LPR group (*n* = 68)	Non-LPR group (*n* = 38)	*p-*value
Sex, female, *n* (%)	29 (43%)	20 (53%)	0.216^a^
Age (years, mean ± SD)	48 ± 10	46 ± 7	0.102^b^
BMI (kg/m^2^, mean ± SD)	23.1 ± 3.0	22.6 ± 2.1	0.279^b^
RSI, mean ± SD	12.0 ± 5.7	3.5 ± 1.8	<0.001^b^
RFS, mean ± SD	9.6 ± 2.7	3.5 ± 1.9	<0.001^b^

LPR, laryngopharyngeal reflux; BMI, body mass index; SD, standard deviation; RSI, reflux symptom index; RFS, reflux finding score.

a The groups were compared using the *χ*^2^ test.

b The groups were compared using the independent samples *t*-test.

### ME-BLI characteristics

**Figure 2:**
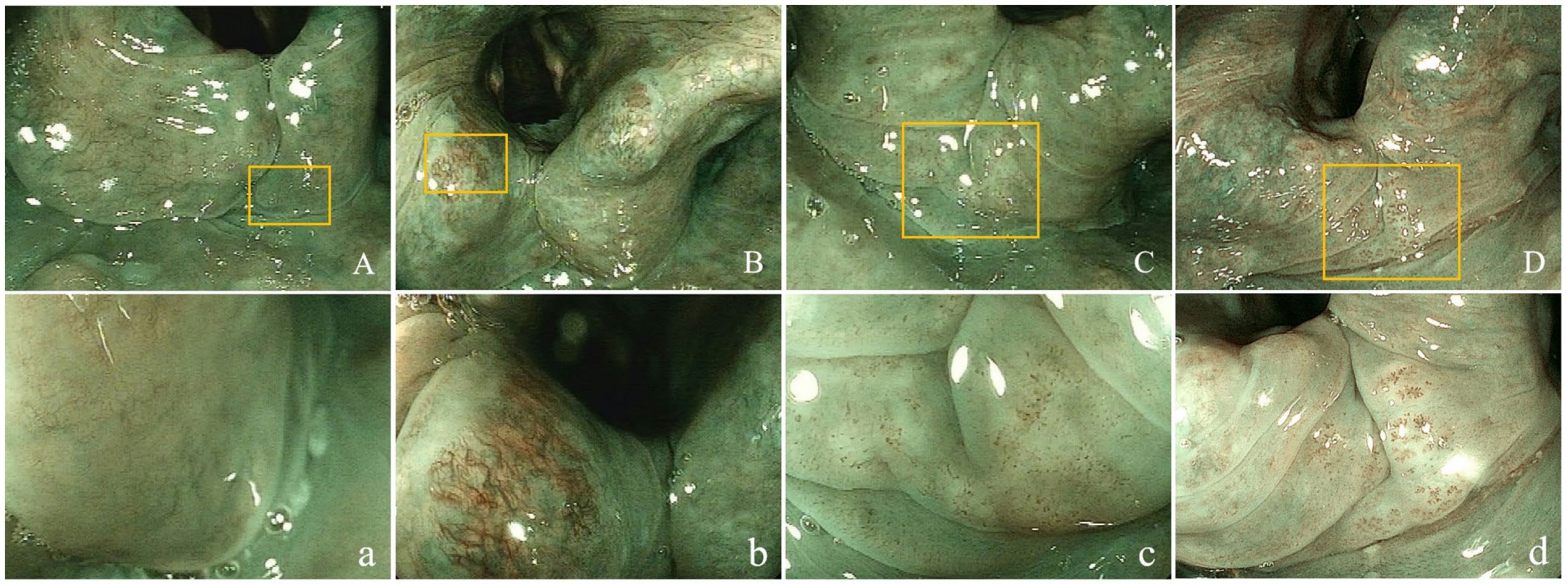
Superficial vascular manifestations of the laryngopharyngeal mucosa. Non-enlarged **(A-D)** and magnified (400×; a- d) target lesions using ME-BLI. Panels a-d are magnifications of the areas marked with yellow rectangles in panels A-D. **(A,a)** ME-BLI Grade 1 images that show no IPCLs and clear intramucosal vessels without dilation, tortuousness, or hyperplasia; **(B,b)** ME-BLI Grade 2 images that show no IPCLs and superficial mucosal blood vessels with hyperplasia, dilation, and tortuousness; **(C,c)** ME-BLI Grade 3 images that show IPCLs and roughly regularly-arranged and sparsely-distributed brown spots in the post-cricoid region and the inter-arytenoid area; **(D,d)** ME-BLI Grade 4 images that show IPCLs and roughly regularly-arranged, densely-distributed, and slightly-dilated brown spots in the post-cricoid region and the inter-arytenoid area. ME-BLI, magnifying endoscopy with blue laser imaging; IPCLs, intraepithelial papillary capillary Loops.

ME-BLI visualized various morphological mucosal microvessel presentations in the laryngopharynx (Figure 2).

The rates of ME-BLI Grades 1, 2, 3, and 4 were 1% (1/68), 9% (6/68), 47% (32/68), and 43% (29/68) in the LPR group and 37% (14/38), 37% (14/38), 16% (6/38), and 11% (4/38) in the non-LPR group, respectively. The overall distribution of the ME-BLI grades differed significantly between the groups (*χ*^2^ = 46.423, *p* < 0.001). The sum of ME-BLI Grades 3 and 4 was 90% (61/68) in the LPR group but only 26% (10/38) in the non-LPR group (*χ*^2^ = 44.291, *p* < 0.001). The brown spots in the mucosa of the post-cricoid, arytenoid, and inter-arytenoid areas were highly specific to LPR (Figures 2C,c,D,d). Based on these results, we defined ME-BLI Grades 3 and 4 as the criterion for LPR positivity, while ME-BLI Grades 1 and 2 were considered LPR-negative. Compared to the clinical reference standard for LPR positivity (RSI >13 points and/or RFS >7 points), this ME-BLI grading classification had a sensitivity of 89.71% (95% CI: 81.54–94.44%), a specificity of 73.68% (95% CI: 59.72–84.03%), a positive predictive value of 85.92% (95% CI: 76.34–92.04%), and a negative predictive value of 80.00% (95% CI: 64.06–90.04%) for LPR diagnosis (Kappa = 0.65, 95% CI: 0.52–0.78, *p* < 0.001).

### Inter-observer agreement

The two observers showed good agreement in ME-BLI grading (ICC = 0.858, *p* < 0.001).

### Reflux-related comorbidities

Upper gastrointestinal endoscopy showed that the LPR group had a higher incidence of cardia relaxation than the non-LPR group (*p* = 0.035; Table 2). No neoplastic diseases of the upper digestive tract were found in either group.

**Table 2:** Comparison of endoscopic findings between the LPR and non-LPR groups.

Endoscopic finding	LPR group (*n* = 68)	non-LPR group (*n* = 38)	*χ*^2^-value	*p-*value
Cardia relaxation	14 (20.588%)	2 (5.263%)	4.467	0.035
Reflux esophagitis	11 (16.176%)	2 (5.363%)	1.779	0.182
Barrett’s esophagus	7 (10.294%)	1 (2.632%)	1.100	0.294
Heterotopic gastric mucosa	5 (7.353%)	0	1.525	0.217
Upper gastrointestinal neoplasms	0	0	—	—

LPR, laryngopharyngeal reflux.

## Discussion

This study applied ME-BLI for LPR diagnosis. The LPR and non-LPR groups differed significantly in their ME-BLI grade distribution (*p* < 0.001). While vascular hyperplasia and dilation (ME-BLI Grade 2) were common in both groups, brown spots (ME-BLI Grades 3 and 4), primarily in the post-cricoid region, were prevalent in the LPR group (90%) and rare in the non-LPR group (26%). IPCLs were frequently observed within these brown spots. The IPCLs exhibit regular papillary or loop-like shapes with a uniform diameter and an orderly arrangement and distribution within the subepithelial lamina propria of normal digestive tract mucosa. Conversely, in the presence of mucosal inflammation, dysplasia, or cancer, IPCLs show abnormalities, such as dilated diameter, distorted morphology, disordered distribution, and neovascularization (11). Their morphological features are closely associated with pathological changes in mucosal tissues, playing a critical role in diagnosing early gastrointestinal lesions (11). IPCLs are also useful in diagnosing reflux diseases. Several studies have utilized NBI laryngoscopy to observe mucosal blood vessels. For instance, He et al. (12) observed features such as “green spots” and increased vascularity in the laryngopharynx of patients with LPR. Similarly, Wu et al. (13) found a higher rate of brown spots in the laryngopharynx of patients with LPR. Ni et al. (10) analyzed and classified the vascular patterns in the laryngopharyngeal mucosa, and Arens et al. (9) proposed relevant descriptive guidelines for vocal cord mucosal vessels. While NBI uses narrow-band filters (415-nm blue light and 540-nm green light), BLI employs laser light sources (410-nm blue laser, 450-nm blue-violet laser). Both light types can aid in visualizing mucosal surface structures and microvasculature, but BLI is advantageous due to its higher light source intensity, brighter images, and clearer details (14). The ME-BLI classification demonstrated good sensitivity (89.71%), specificity (73.68%), positive predictive value (85.92%), and negative predictive value (80.00%) for LPR diagnosis. It also showed good consistency with RSI/RFS diagnosis (Kappa = 0.654, *p* < 0.001). We hypothesized that recurrent reflux triggers chronic pharyngeal irritation, leading to mucosal inflammation. Brown spots occurred most frequently in the post-cricoid region, likely because this is the lowest area in the laryngopharynx, bordering the entrance to the esophagus, making it most vulnerable to reflux. However, the proposed ME-BLI grading system, designed based on vascular changes induced by laryngopharyngeal reflux, remains an exploratory classification. This study lacked histopathological confirmation of the relationship between IPCL changes and tissue inflammation, which warrants further validation.

The RFS system requires complex scoring of multiple items, making it time-consuming. Conversely, ME-BLI diagnosis can be performed rapidly and accurately based on the presence of brown spots. This study demonstrated that ME-BLI was accurate in identifying LPR when compared to the RSI/RFS clinical reference standard. This method is simple and rapid, exhibiting good interobserver agreement (ICC = 0.858, *p* < 0.001), thereby indicating high reproducibility. Furthermore, upper gastrointestinal endoscopy can aid in identifying complications, such as cardia relaxation, and exclude other upper digestive tract diseases, including laryngeal, esophageal, and gastric tumors. We found that the LPR group had a higher rate of cardia relaxation than the non-LPR group (*p* = 0.035). Lower esophageal sphincter relaxation, an important mechanism in LPR pathogenesis, may be improved by anti-reflux surgery (15). ME-BLI can also assist in screening for diseases such as upper digestive tract tumors, particularly in areas where these are prevalent. In summary, ME-BLI is a simple, rapid, effective, and economical method for LPR diagnosis.

Although other methods exist for diagnosing LPR, they all present certain limitations. The knowledge of the RSI score significantly affected doctors’ judgment when assessing patients’ RFS (16). In this study, the RFS evaluators were blinded to the patients’ RSI scores, which helped mitigate such influence. Due to daily fluctuations in the number and characteristics of reflux episodes, even 24-h MII-pH, the gold-standard diagnostic technique, might occasionally produces false negative and false positive results (17). Moreover, the test is expensive and invasive, making it challenging for patients to accept. Furthermore, most primary-level hospitals lack the equipment required to perform the 24-h MII-pH assessment. A survey of otolaryngologists throughout Asia revealed that more than 78% of them never or rarely prescribed 24-h MII-pH (18). Therefore, exploring simple and accurate new technologies is worthwhile.

This study had several limitations. First, this was a pilot study rather than a randomized controlled trial. Second, the study sample was small and originated from a single center; therefore, multi-center verification is recommended. Third, we utilized RSI/RFS as a clinical reference standard instead of the 24-h MII-pH gold-standard test. This choice may have led to an overestimation of the results’ sensitivity. The RSI is a self-reported questionnaire, making it susceptible to subjective influences. Fourth, although we excluded individuals with allergic diseases, acute upper respiratory tract infections, and other conditions when recruiting patients for the study to avoid the impact of factors unrelated to LPR, some other interfering factors may still exist. For example, postnasal drip syndrome may cause symptoms such as cough and pharyngeal discomfort, which could be mistakenly regarded as LPR. This may lead to an increase in false positive results. In the future, efforts could be made to refine the inclusion criteria to enhance the accuracy of the study. For instance, patients with postnasal drip can be excluded based on objective evidence, such as mucus adherence to the nasopharynx observed via nasal endoscopy and sinusitis identified by sinus CT scans. Fifth, the ME-BLI grading system proposed in this study is an exploratory classification that has not been previously validated for LPR. This necessitates further confirmation through histopathological examinations to establish the association between mucosal vascular changes and inflammation. Finally, while we preliminarily analyzed the accuracy of ME-BLI for LPR diagnosis, its efficacy in evaluating treatment outcomes was not assessed. We aim to assess this aspect through a future long-term follow-up study.

## Data Availability

Due to the protection of the subjects’ privacy, the data has not been made public. Requests to access these datasets should be directed to RW, ronglittle2003@163.com.
